# Variation in Management of Extremity Soft-Tissue Sarcoma in Younger vs Older Adults

**DOI:** 10.1001/jamanetworkopen.2021.20951

**Published:** 2021-08-20

**Authors:** Crystal Seldon, Gautam Shrivastava, Abdurrahman Al-Awady, David Asher, Stephen Ramey, Melanie Fernandez, Sarah Dooley, Deukwoo Kwon, Wei Zhao, Neha Goel, Tejan Diwanji, Ty Subhawong, Jonathan Trent, Raphael Yechieli

**Affiliations:** 1 Sylvester Comprehensive Cancer Center, University of Miami, Miami, Florida; 2 Biostatistics and Bioinformatics Shared Resource, Sylvester Comprehensive Cancer Center, University of Miami, Miami, Florida; 3 Department of Radiology, University of Miami, Miami, Florida

## Abstract

**Importance:**

A large proportion of extremity soft-tissue sarcomas (ESS) occur among young adults, yet this group is underrepresented in clinical trials, resulting in limited data on this population. Younger patients present many complex challenges that affect clinical management.

**Objective:**

To investigate variations in treatment management in young adults vs older adults with ESS.

**Design, Setting, and Participants:**

This multicenter retrospective cohort study used the National Cancer Data Base (NCDB) to identify patients 18 years and older with ESS who received definitive treatment (ie, limb-sparing surgery [LSS] or amputation) between 2004 and 2014. Data analysis was conducted in November 2019.

**Exposures:**

Treatment regimen received among young adults (aged 18-39 years) and older adults (≥40 years) after diagnosis with ESS.

**Main Outcomes and Measures:**

To detect unique factors associated with treatment decisions in young adults with ESS, multivariable analyses used logistic regressions for patterns of treatment and their association with demographic factors and tumor characteristics.

**Results:**

Overall, 8953 patients were identified, and among these, 1280 (14.3%) were young adults. From the full cohort, 4796 patients (53.6%) identified as male and 6615 (73.9%) identified as non-Hispanic White. More young adults than older adults underwent amputation (age 18-39 years, 104 of 1280 [8.1%]; age 40-64 years, 217 of 3937 [5.5%]; aged ≥65 years, 199 of 3736 [5.3%]), but the association was not statistically significant (age ≥65 years, odds ratio [OR], 1.49; 95% CI, 1.00-2.23; *P* = .05). Young adults were more likely to receive chemotherapy than older patients (age 40-65 years, OR, 0.52; 95% CI, 0.45-0.60; *P* = .001; ≥65 years, OR, 0.16; 95% CI, 0.12-0.20; *P* = .001). Conversely, young adults were less likely to receive radiation therapy compared with older patients (age 40-65 years, OR, 1.40; 95% CI, 1.22-1.61; *P* = .001; ≥65 years, OR, 1.33; 95% CI, 1.10-1.61; *P* = .003). Unique to younger adults, clinical stage II disease vs stage I and positive surgical margins were not associated with use of radiation therapy (stage II disease: OR, 1.25; 95% CI, 0.81-1.91; *P* = .31; positive surgical margins: OR, 1.43; 95% CI, 0.93-2.22; *P* = .11). White Hispanic young adults were less likely than non-Hispanic White young adults to receive radiation therapy (OR, 0.53; 95% CI, 0.36-0.78; *P* = .002).

**Conclusions and Relevance:**

In this study, young adults with ESS were more likely to receive chemotherapy and less likely to receive radiation therapy than older adults. Further study is warranted to identify the clinical outcomes of these practice disparities.

## Key Points

**Question:**

Is age associated with the treatment regimen given to patients with extremity soft-tissue sarcoma (ESS)?

**Findings:**

In this cohort study of 8953 adults with ESS, there was no significant difference in the likelihood of amputation vs limb-sparing surgery for young adults compared with older adults. However, young adults were less likely to receive radiation therapy and more likely to receive chemotherapy compared with older patients.

**Meaning:**

Further study is warranted to better understand the clinical outcomes associated with these practice disparities.

## Teaser

This cohort study investigates variations in treatment management in young adults vs older adults with extremity soft-tissue sarcomas.

## Introduction

With an estimated incidence of 13 130 new diagnoses in 2020, sarcomas are rare tumors that represent approximately 1% of new cancer diagnoses in the United States.^1^ A diagnosis of sarcoma is not limited to any age, representing 10% of cancers in the adolescent and young adult population.^2^ Sarcomas are grouped based on origin in soft tissues or bone. Although bone sarcomas are less common than soft-tissue sarcomas overall,^2^ the incidence of bone sarcomas peak in the young adult age group.^3^ There are approximately 100 histologic subtypes of sarcoma according to the World Health Organization classification.^4^ Distinct subtypes with higher incidence in the young adult population are osteosarcoma and Ewing sarcoma.^5^ Despite the number of young adults with sarcoma, this group is underrepresented in clinical trials.^6^ Patients with these rare tumors are difficult to study via randomized clinical trials, and the limited data are often extrapolated to young adults. Given poorer outcomes among the young adult population compared with the older adult and pediatric populations,^7^ there is much debate on optimal treatment regimens for this age group. The relatively poor prognosis can be attributed to multiple factors, including location of care, patient education, differing predominant histology, poor clinical trial participation, and a lack of a designated care system.^8,9,10^ Young adults present many complex challenges that affect clinical management. To further identify practice and treatment disparities among the young adult group, the National Cancer Database (NCDB) was used to identify patients 18 years and older with extremity soft-tissue sarcoma (ESS) diagnosed between 2004 and 2014 and treated definitively with limb-sparing surgery (LSS) or amputation. We hypothesized that the NCDB could detect unique factors that are associated with treatment decisions in young adults with ESS.

## Methods

This retrospective cohort followed the Strengthening the Reporting of Observational Studies in Epidemiology (STROBE) reporting guideline for cohort studies and was deemed exempt from institutional review board approval per the Human Subjects Research Office of the University of Miami. Informed consent was waived because the data were deidentified. The NCDB is an oncology-based database populated from more than 1500 Commission on Cancer (CoC) institutions.^11,12^ These hospitals are recognized by the American College of Surgeons CoC as cancer programs that fulfill the requirements for accreditation. This deidentified registry includes information on patient demographic characteristics, tumor characteristics, treatment methods, and survival outcomes. Neither the CoC-accredited institutions nor the NCDB are responsible for the present analysis and conclusions made by the presenting authors. The NCDB was used to identify patients 18 years and older with ESS diagnosed between 2004 and 2014 and treated definitively with LSS or amputation. Exclusion criteria included patients without follow-up pertaining to vital status and patients who were treated with nonstandard radiation therapy (RT) modalities, such as electrons. We defined the young adult population as those aged 18 to 39 years. Overall, 8953 patients were identified. The following variables were obtained from the NCDB: history of RT, history of chemotherapy, surgical treatment type, sex, race and ethnicity (White non-Hispanic, White Hispanic, Black, and other or unknown), insurance status, median income in 2012, the percentage of the population in the patient’s zip code without a high school degree, living location, distance from living location to treating facility, presence of comorbidities, if a transition in care occurred, tumor grade, date of diagnosis, primary site of tumor, depth of extension, surgical margin status, size of tumor, and clinical tumor stage. Study variables were defined similarly to a previous study from our institution.^13^ We examined race and ethnicity data because previous studies have indicated racial/ethnic disparities in the incidence of soft-tissue sarcoma. Median income and high school graduation rates were derived from the 2012 American Community Survey. Urban/rural status was defined using the 2013 files published by the US Department of Agriculture Economic Research Service. The American Joint Commission on Cancer sixth edition was used for patients diagnosed between 2004 and 2009, and the seventh edition was used for patients diagnosed between 2010 and 2014.^13^

### Statistical Analysis

Descriptive summary statistics for the baseline clinicopathological and socioeconomic variables were reported, and χ^2^ tests were used to compare the characteristics. Factors associated with treatment selection were evaluated using univariable and multivariable logistic regression models. Analysis was conducted by each age group and overall. Odds ratios (ORs) and corresponding 95% CIs and *P* values were reported. All tests were 2-sided, and statistical significance was set at *P* < .05. Statistical software SAS version 9.4 (SAS Institute) was used for the analysis.

## Results

### Demographic and Clinical Characteristics

Overall, 8953 patients were identified, and among these, 1280 (14.3%) were young adults. Of all the age groups, 4796 patients (53.6%) identified as male, and 6615 (73.9%) identified as non-Hispanic White. Overall, 2949 patients (32.9%) had a median household income of or greater than $63 000 in 2012, and 4500 (50.3%) lived in metropolitan locations (ie, population >250 000). Most patients received RT (5847 [65.3%]) and no chemotherapy (6689 [74.7%]). Most patients received LSS with or without RT (8433 [94.2%]). Of the 5847 patients who received RT, 4046 (69.2%) received treatment postoperatively. Overall, 6765 patients (75.6%) presented with primary tumors of the lower limb. Most tumors had a deep depth of extension (5873 [65.8%]) and were poorly differentiated (4698 [52.5%]). Demographic and clinical characteristics of the patient population appear in in Table 1.

**Table 1.** Demographic and Clinical Characteristics of Patients With Extremity Soft-Tissue Sarcoma in the National Cancer Database

Characteristic	Patients, No. (%)				*P* value^a^
	Total (N = 8953)	Age, y			
		18-39 (n = 1280)	40-64 (n = 3937)	≥65 (n = 3736)	
Radiation					
No RT	3106 (34.7)	521 (40.7)	1218 (30.9)	1367 (36.6)	<.001
RT	5847 (65.3)	759 (59.3)	2719 (69.1)	2369 (63.4)	
RT type					
No RT	3106 (34.7)	521 (40.7)	1218 (30.9)	1367 (36.6)	<.001
Preoperative RT	1801 (20.1)	235 (18.4)	891 (22.6)	675 (18.1)	
Postoperative RT	4046 (45.2)	524 (40.9)	1828 (46.4)	1694 (45.3)	
Chemotherapy					
None	6689 (74.7)	739 (57.7)	2667 (67.7)	3283 (87.9)	<.001
Given	2012 (22.5)	504 (39.4)	1161 (29.5)	347 (9.3)	
Unknown	252 (2.8)	37 (2.9)	109 (2.8)	106 (2.8)	
Treatment					
Amputation	520 (5.8)	104 (8.1)	217 (5.5)	199 (5.3)	<.001
LSS with or without RT	8433 (94.2)	1176 (91.9)	3720 (94.5)	3537 (94.7)	
Sex					
Male	4796 (53.6)	708 (55.3)	2239 (56.9)	1849 (49.5)	<.001
Female	4157 (46.4)	572 (44.7)	1698 (43.1)	1887 (50.5)	
Race/ethnicity					
White Non-Hispanic	6615 (73.9)	780 (60.9)	2855 (72.5)	2980 (79.8)	<.001
White Hispanic	557 (6.2)	166 (13.0)	260 (6.6)	131 (3.5)	
Black	921 (10.3)	192 (15.0)	464 (11.8)	265 (7.1)	
Other or unknown^b^	860 (9.6)	142 (11.1)	358 (9.1)	360 (9.6)	
Insurance status					
Not insured	375 (4.2)	125 (9.8)	229 (5.8)	21 (0.6)	<.001
Private insurance	4355 (48.6)	873 (68.2)	3020 (76.7)	462 (12.4)	
Medicaid	524 (5.9)	198 (15.5)	270 (6.9)	56 (1.5)	
Medicare	3425 (38.3)	38 (3.0)	267 (6.8)	3120 (83.5)	
Other or unknown	274 (3.1)	46 (3.6)	151 (3.8)	77 (2.1)	
Median income in patient’s zip code in 2012, $					
<38 000	1460 (16.3)	233 (18.2)	657 (16.7)	570 (15.3)	.009
38 000-$47 999	2033 (22.7)	278 (21.7)	873 (22.2)	882 (23.6)	
48 000-$62 999	2369 (26.5)	347 (27.1)	984 (25.0)	1038 (27.8)	
≥63 000	2949 (32.9)	405 (31.6)	1361 (34.6)	1183 (31.7)	
Unknown	142 (1.6)	17 (1.3)	62 (1.6)	63 (1.7)	
Non–high school graduates, % of population in patient’s zip code					
≥21	1466 (16.4)	251 (19.6)	694 (17.6)	521 (13.9)	<.001
13-20.9	2140 (23.9)	300 (23.4)	903 (22.9)	937 (25.1)	
7-12.9	2911 (32.5)	419 (32.7)	1260 (32.0)	1232 (33.0)	
<7	2308 (25.8)	296 (23.1)	1027 (26.1)	985 (26.4)	
Unknown	128 (1.4)	14 (1.1)	53 (1.3)	61 (1.6)	
Living location					
Metropolitan^c^	4500 (50.3)	668 (52.2)	2055 (52.2)	1777 (47.6)	<.001
Smaller metropolitan^c^	2699 (30.1)	397 (31.0)	1125 (28.6)	1177 (31.5)	
Urban	1289 (14.4)	164 (12.8)	558 (14.2)	567 (15.2)	
Rural	139 (1.6)	11 (0.9)	64 (1.6)	64 (1.7)	
Unknown	326 (3.6)	40 (3.1)	135 (3.4)	151 (4.0)	
Distance to reporting facility, miles					
≤10	3145 (35.1)	404 (31.6)	1357 (34.5)	1384 (37.0)	.004
11-20	1652 (18.5)	250 (19.5)	758 (19.3)	644 (17.2)	
21-50	1798 (20.1)	258 (20.2)	815 (20.7)	725 (19.4)	
>50	2232 (24.9)	355 (27.7)	952 (24.2)	925 (24.8)	
Unknown	126 (1.4)	13 (1.0)	55 (1.4)	58 (1.6)	
Comorbidity, No.					
0	7360 (82.2)	1214 (94.8)	3374 (85.7)	2772 (74.2)	<.001
1	1303 (14.6)	61 (4.8)	471 (12.0)	771 (20.6)	
2	290 (3.2)	5 (0.4)	92 (2.3)	193 (5.2)	
Transition in care					
No	1358 (15.2)	212 (16.6)	571 (14.5)	575 (15.4)	<.001
Yes	4851 (54.2)	683 (53.4)	2174 (55.2)	1994 (53.4)	
Unknown	2744 (30.6)	385 (30.1)	1192 (30.3)	1167 (31.2)	
Primary site					
Upper limb	2188 (24.4)	338 (26.4)	878 (22.3)	972 (26.0)	<.001
Lower limb	6765 (75.6)	942 (73.6)	3059 (77.7)	2764 (74.0)	
Grade					
MD	1125 (12.6)	229 (17.9)	519 (13.2)	377 (10.1)	<.001
PD	4698 (52.5)	666 (52.0)	2037 (51.7)	1995 (53.4)	
UD	3130 (35.0)	385 (30.1)	1381 (35.1)	1364 (36.5)	
Tumor size, cm					
≤5.00	2610 (29.2)	409 (32.0)	1117 (28.4)	1084 (29.0)	<.001
5.01-10.00	3288 (36.7)	480 (37.5)	1392 (35.4)	1416 (37.9)	
10.01-15.00	1690 (18.9)	240 (18.8)	774 (19.7)	676 (18.1)	
>15.00	1365 (15.2)	151 (11.8)	654 (16.6)	560 (15.0)	
Clinical tumor stage					
I	1935 (21.6)	301 (23.5)	824 (20.9)	810 (21.7)	.34
II	4978 (55.6)	704 (55.0)	2210 (56.1)	2064 (55.2)	
Unknown	2040 (22.8)	275 (21.5)	903 (22.9)	862 (23.1)	
Depth of extension					
Superficial	2324 (26.0)	301 (23.5)	956 (24.3)	1067 (28.6)	<.001
Deep	5873 (65.6)	850 (66.4)	2657 (67.5)	2366 (63.3)	

Abbreviations: LSS, limb-sparing surgery; MD, moderately differentiated; PD, poorly differentiated; RT, radiation therapy; UD, undifferentiated.

^a^ *P* value derived from χ^2^ test.

^b^ Other races/ethnicities included American Indian, Aleutian, or Eskimo; Chinese; Japanese; Filipino; Hawaiian; Korean; Vietnamese; Laotian; Hmong; Kampuchean; Thai; Asian Indian or Pakistani, not otherwise specified; Asian Indian; Pakistani; Micronesian, not otherwise specified; Chamorran; Guamanian, not otherwise specified; Polynesian, not otherwise specified; Tahitian, Samoan, Tongan, Melanesian, not otherwise specified; Fiji Islander; New Guinean; other Asian, including Asian, not otherwise specified, and Oriental, not otherwise specified; and Pacific Islander, not otherwise specified.

^c^ Metropolitan was defined as having a population of 250 000 or greater; smaller metropolitan, 20 000 to less than 250 000.

Young adult patients, vs those aged 65 years and older, were more likely to be medically uninsured (125 of 1280 [9.8%] vs 21 of 3736 [0.6%]), have a median income of less than $38 000 (233 [18.2%] vs 570 [15.3%]), and live in an area with a higher percentage of residents without a high school education (≥21% of population without high school degree: 251 [19.6%] vs 521 [13.9%]). Young adults vs those aged 65 years and older were proportionally less likely to identify as White non-Hispanic (780 [60.9%] vs 2980 [79.8%]), with greater percentages of Black (192 [15.0%] vs 265 [7.1%]) and White Hispanic (166 [13.0%] vs 131 [3.5%]) patients. More young adults underwent amputation than any other age group (age 18-39 years, 104 [8.1%]; age 40-64 years, 217 of 3937 [5.5%]; aged ≥65 years, 199 [5.3%]).

Univariable analysis on RT vs non-RT was performed, and the results are shown in Table 2. Among other factors, White Hispanic race/ethnicity, private insurance, median income of at least $63 000, and living more than 50 miles from the reporting facility were associated with receipt of RT in the young adult cohort.

**Table 2.** Univariable Logistic Regression: Association of Selected Factors With Receipt of RT

Factor	Age 18-39 y (n = 1280)		Age 40-64 y (n = 3937)		Age ≥65 y (n = 3736)		All (N = 8953)	
	OR (95% CI)	*P* value	OR (95% CI)	*P* value	OR (95% CI)	*P* value	OR (95% CI)	*P* value
Sex								
Male	1 [Reference]	NA	1 [Reference]	NA	1 [Reference]	NA	1 [Reference]	NA
Female	0.78 (0.62-0.97)	.03	1.05 (0.91-1.20)	.52	0.92 (0.80-1.05)	.21	0.93 (0.86-1.02)	.12
Race/ethnicity								
White Non-Hispanic	1 [Reference]	NA	1 [Reference]	NA	1 [Reference]	NA	1 [Reference]	NA
White Hispanic	0.50 (0.36-0.70)	.001	0.80 (0.61-1.05)	.10	0.77 (0.54-1.10)	.15	0.69 (0.58-0.83)	.001
Black	0.74 (0.54-1.02)	.07	0.90 (0.73-1.11)	.34	1.07 (0.82-1.39)	.64	0.93 (0.80-1.07)	.30
Other or unknown^a^	0.72 (0.50-1.04)	.08	0.89 (0.70-1.12)	.32	1.03 (0.82-1.30)	.79	0.91 (0.78-1.05)	.21
Insurance status								
Not insured	1 [Reference]	NA	1 [Reference]	NA	1 [Reference]	NA	1 [Reference]	NA
Private insurance	2.10 (1.44-3.06)	<.001	1.54 (1.17-2.04)	.002	2.45 (1.02-5.91)	.045	1.83 (1.48-2.27)	.001
Medicaid	1.28 (0.82-2.01)	.28	1.21 (0.84-1.74)	.31	1.70 (0.62-4.67)	.30	1.21 (0.92-1.58)	.17
Medicare	1.37 (0.66-2.83)	.40	0.97 (0.67-1.39)	.85	1.83 (0.77-4.32)	.17	1.35 (1.09-1.67)	.006
Other or unknown	2.82 (1.37-5.79)	.005	1.32 (0.86-2.04)	.20	3.36 (1.23-9.13)	.02	1.92 (1.38-2.67)	<.001
Median income in patient’s zip code in 2012, $								
<38 000	1 [Reference]	NA	1 [Reference]	NA	1 [Reference]	NA	1 [Reference]	NA
48 000-62 999	1.02 (0.76-1.37)	.90	0.91 (0.76-1.09)	.32	0.92 (0.77-1.10)	.35	0.92 (0.82-1.03)	.17
38 000-47 999	0.76 (0.56-1.04)	.09	0.93 (0.77-1.12)	.44	0.88 (0.73-1.06)	.17	0.88 (0.78-0.99)	.03
≥63 000	0.58 (0.42-0.81)	.001	0.82 (0.67-1.00)	.045	0.76 (0.62-0.93)	.008	0.74 (0.65-0.85)	.001
Unknown	1.08 (0.39-2.98)	.88	1.02 (0.58-1.78)	.95	0.57 (0.34-0.95)	.03	0.78 (0.55-1.10)	.16
Non–high school graduates, % of population in patient’s zip code								
<7	1 [Reference]	NA	1 [Reference]	NA	1 [Reference]	NA	1 [Reference]	NA
7-12.9	1.17 (0.86-1.59)	.32	0.91 (0.76-1.08)	.28	0.89 (0.74-1.06)	.18	0.93 (0.83-1.04)	.20
13-20.9	0.99 (0.72-1.38)	.97	0.92 (0.76-1.12)	.43	0.88 (0.73-1.06)	.18	0.91 (0.80-1.03)	.12
≥21	0.70 (0.50-0.98)	.04	0.79 (0.65-0.98)	.03	0.75 (0.60-0.93)	.01	0.76 (0.66-0.87)	.001
Unknown	1.21 (0.40-3.70)	.74	1.04 (0.56-1.91)	.91	0.56 (0.34-0.95)	.03	0.78 (0.54-1.12)	.18
Living location								
Metropolitan^b^	1 [Reference]	NA	1 [Reference]	NA	1 [Reference]	NA	1 [Reference]	NA
Smaller metropolitan^b^	0.87 (0.67-1.12)	.28	0.85 (0.73-0.99)	.04	1.05 (0.90-1.22)	.57	0.92 (0.84-1.02)	.12
Urban	0.79 (0.56-1.12)	.19	0.85 (0.70-1.04)	.11	1.15 (0.95-1.41)	.16	0.96 (0.84-1.09)	.54s
Rural	0.53 (0.16-1.76)	.30	0.80 (0.47-1.35)	.40	0.98 (0.59-1.65)	.95	0.86 (0.61-1.22)	.39
Unknown	1.18 (0.61-2.31)	.62	1.19 (0.80-1.78)	.38	0.76 (0.54-1.06)	.11	0.94 (0.74-1.19)	.62
Distance to reporting facility, miles								
≤10	1 [Reference]	NA	1 [Reference]	NA	1 [Reference]	NA	1 [Reference]	NA
11-20	0.74 (0.54-1.03)	.08	0.81 (0.67-0.99)	.04	0.86 (0.71-1.05)	.14	0.83 (0.73-0.94)	.004
21-50	0.82 (0.60-1.14)	.24	0.71 (0.59-0.86)	<.001	0.98 (0.81-1.18)	.82	0.84 (0.74-0.95)	.005
>50	0.57 (0.43-0.76)	<.001	0.50 (0.42-0.60)	.001	0.66 (0.56-0.79)	.001	0.58 (0.52-0.65)	.001
Unknown	0.85 (0.27-2.64)	.78	0.81 (0.45-1.48)	.50	0.57 (0.34-0.97)	.04	0.69 (0.48-1.00)	.05
Comorbidities, No.								
0	1 [Reference]	NA	1 [Reference]	NA	1 [Reference]	NA	1 [Reference]	NA
1	1.14 (0.67-1.93)	.63	0.82 (0.67-1.00)	.05	0.78 (0.66-0.92)	.003	0.82 (0.72-0.92)	<.001
2	0.46 (0.08-2.76)	.40	0.64 (0.42-0.98)	.04	0.73 (0.54-0.98)	.04	0.70 (0.55-0.89)	.003
Transition in care								
No	1 [Reference]	NA	1 [Reference]	NA	1 [Reference]	NA	1 [Reference]	NA
Yes	1.30 (0.95-1.78)	.10	1.26 (1.04-1.54)	.02	1.67 (1.38-2.01)	.001	1.44 (1.27-1.63)	.001
Unknown	0.91 (0.65-1.28)	.59	1.09 (0.88-1.35)	.42	1.19 (0.98-1.46)	.09	1.11 (0.97-1.27)	.12
Primary site								
Upper limb	1 [Reference]	NA	1 [Reference]	NA	1 [Reference]	NA	1 [Reference]	NA
Lower limb	1.09 (0.85-1.41)	.48	1.06 (0.90-1.24)	.49	0.91 (0.78-1.06)	.23	1.01 (0.91-1.12)	.84
Grade								
MD	1 [Reference]	NA	1 [Reference]	NA	1 [Reference]	NA	1 [Reference]	NA
PD	1.08 (0.80-1.47)	.62	1.17 (0.96-1.44)	.12	1.21 (0.97-1.51)	.10	1.16 (1.02-1.33)	.03
UD	0.92 (0.66-1.28)	.62	1.19 (0.96-1.48)	.11	1.28 (1.01-1.61)	.04	1.18 (1.02-1.36)	.02
Tumor size, cm								
≤5.00	1 [Reference]	NA	1 [Reference]	NA	1 [Reference]	NA	1 [Reference]	NA
5.01-10.00	1.70 (1.30-2.22)	<.001	1.84 (1.56-2.18)	.001	1.47 (1.25-1.73)	.001	1.64 (1.48-1.83)	.001
10.01-15.00	1.92 (1.38-2.67)	<.001	1.77 (1.45-2.16)	.001	1.71 (1.40-2.09)	.001	1.78 (1.56-2.02)	.001
>15.00	1.36 (0.93-1.99)	.11	1.53 (1.25-1.88)	.001	1.29 (1.04-1.59)	.02	1.43 (1.25-1.64)	.001
Clinical tumor stage								
I	1 [Reference]	NA	1 [Reference]	NA	1 [Reference]	NA	1 [Reference]	NA
II	1.63 (1.24-2.14)	<.001	1.99 (1.69-2.36)	.001	1.60 (1.35-1.89)	.001	1.76 (1.58-1.97)	.001
Unknown	1.27 (0.92-1.77)	.152	1.43 (1.17-1.74)	<.001	1.15 (0.95-1.40)	.16	1.29 (1.13-1.46)	<.001
Depth of extension								
Superficial	1 [Reference]	NA	1 [Reference]	NA	1 [Reference]	NA	1 [Reference]	NA
Deep	1.85 (1.42-2.42)	.001	1.64 (1.40-1.91)	.001	1.30 (1.12-1.51)	<.001	1.51 (1.37-1.67)	.001
Unknown	1.20 (0.80-1.82)	.38	1.07 (0.83-1.39)	.59	0.97 (0.74-1.25)	.79	1.03 (0.87-1.22)	.72
Surgical margin								
Negative	1 [Reference]	NA	1 [Reference]	NA	1 [Reference]	NA	1 [Reference]	NA
Positive	1.33 (0.89-2.01)	.17	1.33 (1.06-1.65)	.01	1.26 (1.05-1.50)	.01	1.27 (1.12-1.45)	<.001
Unknown	0.75 (0.43-1.29)	.30	1.39 (0.93-2.07)	.11	1.22 (0.86-1.73)	.26	1.15 (0.91-1.45)	.24
Age, y								
18-39	NA	NA	NA	NA	NA	NA	1 [Reference]	NA
40-64	NA	NA	NA	NA	NA	NA	1.53 (1.35-1.75)	.001
≥65	NA	NA	NA	NA	NA	NA	1.19 (1.04-1.35)	.009

Abbreviations: MD, moderately differentiated; NA, not applicable; OR, odds ratio; PD, poorly differentiated; RT, radiation therapy; UD, undifferentiated.

^a^ Other races/ethnicities included American Indian, Aleutian, or Eskimo; Chinese; Japanese; Filipino; Hawaiian; Korean; Vietnamese; Laotian; Hmong; Kampuchean; Thai; Asian Indian or Pakistani, not otherwise specified; Asian Indian; Pakistani; Micronesian, not otherwise specified; Chamorran; Guamanian, not otherwise specified; Polynesian, not otherwise specified; Tahitian, Samoan, Tongan, Melanesian, not otherwise specified; Fiji Islander; New Guinean; other Asian, including Asian, not otherwise specified, and Oriental, not otherwise specified; and Pacific Islander, not otherwise specified.

^b^ Metropolitan was defined as having a population of 250 000 or greater; smaller metropolitan, 20 000 to less than 250 000.

### Multivariable Analysis of RT

In the young adult age group, sex, Black or other race, nonprivate insurance, income status, educational status, living location, comorbidities, transitions in care, primary site and grade of tumor, clinical tumor stage, and surgical margin status were not prognostic for treatment with RT (Table 3). Young adults who identified as White Hispanic vs those who identified as White non-Hispanic were less likely to receive RT (OR, 0.53; 95% CI, 0.36-0.78; *P* = .002). Young adult patients with private insurance (OR, 1.80; 95% CI, 1.19-2.72; *P* = .006), deep depth of extension of the tumor (OR, 1.86; 95% CI, 1.38-2.51; *P* = .001), and tumors measuring 10.01 to 15.00 cm (OR, 1.60; 95% CI, 1.02-2.52; *P* = .04) were associated with a greater likelihood of receiving RT. The ORs for the receipt of RT that were significant in 1 age group were in the same direction (ie, >1 or <1) in the other age groups, even if they were not significant, for all factors except comorbidity score of 1 (OR, <1 for age 40-64 years and ≥65 years, but >1 for young adult group), lower limb primary site (<1 for age 40-64 years and ≥65 years, but >1 for young adult group), and undifferentiated grade (>1 for age 40-64 years and ≥65 years, but >1 for young adult group). Unique to young adults, clinical stage II disease compared with stage I and positive surgical margins were not associated with use of RT (clinical stage II: OR, 1.25; 95% CI, 0.81-1.91; *P* = .31; positive surgical margins: OR, 1.43; 95% CI, 0.93-2.22; *P* = .11). For all ages, deep tumor extension (OR, 1.37; 95% CI, 1.22-1.53; *P* = .001) and tumor size (5.01-10.00 cm: OR, 1.30; 95% CI, 1.12-1.51; *P* = .001) were associated with the receipt of RT. Factors associated with increased or decreased RT use by age group appear in Table 4.

**Table 3.** Multivariable Logistic Regression: Association of Selected Factors With Receipt of RT by Age Group and Overall

Category	Age 18-39 y (n = 1280)		Age 40-64 y (n = 3937)		Age ≥65 y (n = 3736)		All (N = 8953)	
	OR (95% CI)	*P* value	OR (95% CI)	*P* value	OR (95% CI)	*P* value	OR (95% CI)	*P* value
Sex								
Male	1 [Reference]	NA	1 [Reference]	NA	1 [Reference]	NA	1 [Reference]	NA
Female	0.81 (0.64-1.03)	.09	1.06 (0.92-1.22)	.42	0.95 (0.83-1.09)	.47	0.98 (0.89-1.07)	.62
Race/ethnicity								
White non-Hispanic	1 [Reference]	NA	1 [Reference]	NA	1 [Reference]	NA	1 [Reference]	NA
White Hispanic	0.53 (0.36-0.78)	.002	0.73 (0.54-0.98)	.04	0.80 (0.55-1.17)	.25	0.71 (0.58-0.87)	<.001
Black	0.80 (0.56-1.15)	.23	0.86 (0.68-1.08)	.20	1.14 (0.86-1.51)	.36	0.95 (0.81,1.11)	.51
Other or unknown^a^	0.68 (0.46-1.00)	.05	0.83 (0.65-1.06)	.14	0.98 (0.78-1.25)	.89	0.87 (0.74-1.01)	.07
Insurance status								
Not insured	1 [Reference]	NA	1 [Reference]	NA	1 [Reference]	NA	1 [Reference]	NA
Private Insurance	1.80 (1.19-2.72)	.006	1.60 (1.20-2.15)	.002	2.00 (0.79-5.04)	.14	1.68 (1.34-2.11)	.001
Medicaid	1.15 (0.71-1.84)	.57	1.28 (0.87-1.86)	.21	1.44 (0.50-4.14)	.49	1.22 (0.92-1.60)	.17
Medicare	1.30 (0.59-2.84)	.51	0.99 (0.68-1.45)	.97	1.56 (0.63-3.86)	.34	1.19 (0.92-1.55)	.18
Other or unknown	3.59 (1.66-7.78)	.001	1.38 (0.88-2.16)	.16	2.99 (1.05-8.55)	.04	1.92 (1.36-2.70)	<.001
Median income in patient’s zip code in 2012, $								
>63 000	1 [Reference]	NA	1 [Reference]	NA	1 [Reference]	NA	1 [Reference]	NA
48 000-62 999	1.01 (0.69-1.46)	.98	1.00 (0.81-1.23)	.96	0.91 (0.74-1.12)	.36	0.96 (0.84-1.10)	.58
38 000-47 999	0.76 (0.50-1.17)	.22	1.07 (0.83-1.37)	.61	0.85 (0.67-1.09)	.20	0.92 (0.79-1.08)	.33
<38 000	0.68 (0.41-1.13)	.14	0.97 (0.72-1.31)	.84	0.76 (0.56-1.03)	.07	0.83 (0.68-1.01)	.06
Unknown	1.05 (0.10-11.42)	.97	0.72 (0.18-2.93)	.65	1.00 (0.06-16.21)	>.99	0.92 (0.30-2.87)	.89
Non–high school graduates, % of population in patient’s zip code								
<7	1 [Reference]	NA	1 [Reference]	NA	1 [Reference]	NA	1 [Reference]	NA
7-12.9	1.38 (0.96-1.98)	.09	0.96 (0.78-1.17)	.66	0.95 (0.78-1.16)	.61	1.00 (0.88-1.14)	.97
13-20.9	1.40 (0.91-2.15)	.13	0.96 (0.75-1.23)	.75	0.95 (0.75-1.20)	.68\	1.02 (0.87-1.19)	.85
≥21	1.23 (0.74-2.03)	.42	0.90 (0.67-1.20)	.47	0.81 (0.61-1.09)	.17	0.90 (0.75-1.09)	.30
Unknown	1.20 (0.01-229.42)	.95	1.30 (0.08-20.20)	.85	0.26 (0.01-10.57)	.47	0.70 (0.10-4.72)	.71
Living location								
Metropolitan^b^	1 [Reference]	NA	1 [Reference]	NA	1 [Reference]	NA	1 [Reference]	NA
Smaller metropolitan^b^	1.00 (0.73-1.37)	.99	1.08 (0.90-1.30)	.43	1.33 (1.11-1.59)	.002	1.17 (1.04-1.32)	.008
Urban	1.15 (0.74-1.79)	.53	1.31 (1.02-1.69)	.04	1.92 (1.49-2.47)	.001	1.51 (1.29-1.79)	.001
Rural	0.78 (0.22-2.84)	.71	1.52 (0.86-2.67)	.15	1.77 (1.02-3.08)	.04	1.55 (1.06-2.25)	.02
Unknown	1.40 (0.61-3.21)	.43	1.44 (0.87-2.39)	.16	0.95 (0.62-1.48)	.84	1.17 (0.86-1.58)	.31
Distance to reporting facility, miles								
≤10	1 [Reference]	NA	1 [Reference]	NA	1 [Reference]	NA	1 [Reference]	NA
11-20	0.61 (0.43-0.87)	.007	0.76 (0.61-0.93)	.009	0.75 (0.61-0.92)	.005	0.73 (0.64-0.84)	.001
21-50	0.59 (0.41-0.84)	.004	0.61 (0.50-0.75)	.001	0.78 (0.64-0.96)	.02	0.68 (0.60-0.78)	.001
>50	0.39 (0.27-0.56)	.001	0.38 (0.31-0.47)	.001	0.43 (0.35-0.53)	.001	0.40 (0.35-0.46)	.001
Unknown	0.32 (0.00-42.37)	.65	0.53 (0.06-5.02)	.58	2.02 (0.16-26.18)	.59	0.76 (0.16-3.63)	.73
Comorbidities, No.								
0	1 [Reference]	NA	1 [Reference]	NA	1 [Reference]	NA	1 [Reference]	NA
1	1.20 (0.68-2.11)	.53	0.88 (0.71-1.09)	.23	0.78 (0.65-0.92)	.003	0.82 (0.72-0.93)	.003
2	0.60 (0.08-4.32)	.62	0.71 (0.45-1.10)	.12	0.76 (0.56-1.04)	.08	0.74 (0.58-0.95)	.02
Transition in care								
No	1 [Reference]	NA	1 [Reference]	NA	1 [Reference]	NA	1 [Reference]	NA
Yes	1.37 (0.97-1.93)	.07	1.45 (1.18-1.79)	<.001	1.87 (1.53-2.28)	.001	1.61 (1.41-1.84)	.001
Unknown	0.83 (0.57-1.21)	.34	1.08 (0.86-1.35)	.53	1.16 (0.94-1.44)	.17	1.07 (0.93-1.23)	.34
Primary site								
Upper limb	1 [Reference]	NA	1 [Reference]	NA	1 [Reference]	NA	1 [Reference]	NA
Lower limb	1.03 (0.78-1.35)	.86	0.93 (0.78-1.10)	.38	0.84 (0.71-0.99)	.04	0.91 (0.81-1.01)	.07
Grade								
MD	1 [Reference]	NA	1 [Reference]	NA	1 [Reference]	NA	1 [Reference]	NA
PD	1.03 (0.73-1.44)	.88	1.16 (0.93-1.44)	.18	1.20 (0.95-1.52)	.13	1.14 (0.99-1.31)	.07
UD	0.87 (0.61-1.25)	.46	1.22 (0.97-1.53)	.09	1.29 (1.01-1.64)	.04	1.17 (1.01-1.36)	.04
Tumor size, cm								
≤5.00	1 [Reference]	NA	1 [Reference]	NA	1 [Reference]	NA	1 [Reference]	NA
5.01-10.00	1.44 (0.97-2.14)	.07	1.28 (1.02-1.62)	.03	1.25 (1.00-1.56)	.05	1.30 (1.12-1.51)	<.001
10.01-15.00	1.60 (1.02-2.52)	.04	1.21 (0.93-1.58)	.16	1.49 (1.14-1.94)	.004	1.39 (1.17-1.65)	<.001
>15.00	1.14 (0.69-1.86)	.61	1.07 (0.82-1.40)	.64	1.09 (0.83-1.42)	.53	1.10 (0.92-1.31)	.29
Clinical tumor stage								
I	1 [Reference]	NA	1 [Reference]	NA	1 [Reference]	NA	1 [Reference]	NA
II	1.25 (0.81-1.91)	.31	1.66 (1.30-2.13)	.001	1.32 (1.04-1.69)	.02	1.45 (1.24-1.71)	.001
Unknown	1.10 (0.74-1.64)	.65	1.24 (0.98-1.56)	.07	0.98 (0.78-1.24)	.86	1.11 (0.95-1.29)	.19
Depth of extension								
Superficial	1 [Reference]	NA	1 [Reference]	NA	1 [Reference]	NA	1 [Reference]	NA
Deep	1.86 (1.38-2.51)	.001	1.46 (1.23-1.74)	.001	1.21 (1.02-1.43)	.03	1.37 (1.22-1.53)	.001
Unknown	1.42 (0.92-2.21)	.12	1.06 (0.81-1.39)	.68	0.92 (0.70-1.20)	.53	1.03 (0.86-1.22)	.77
Surgical margin								
Negative	1 [Reference]	NA	1 [Reference]	NA	1 [Reference]	NA	1 [Reference]	NA
Positive	1.43 (0.93-2.22)	.11	1.31 (1.04-1.64)	.02	1.27 (1.06-1.53)	.01	1.28 (1.12-1.47)	<.001
Unknown	0.69 (0.38-1.25)	.23	1.24 (0.82-1.87)	.31	1.08 (0.75-1.55)	.69	1.07 (0.84-1.36)	.60
Age, y								
18-39	NA	NA	NA	NA	NA	NA	1 [Reference]	NA
40-64	NA	NA	NA	NA	NA	NA	1.40 (1.22-1.61)	.001
≥65	NA	NA	NA	NA	NA	NA	1.33 (1.10-1.61)	.003

Abbreviations: MD, moderately differentiated; NA, not applicable; OR, odds ratio; PD, poorly differentiated; RT, radiation therapy; UD, undifferentiated.

^a^ Other races/ethnicities included American Indian, Aleutian, or Eskimo; Chinese; Japanese; Filipino; Hawaiian; Korean; Vietnamese; Laotian; Hmong; Kampuchean; Thai; Asian Indian or Pakistani, not otherwise specified; Asian Indian; Pakistani; Micronesian, not otherwise specified; Chamorran; Guamanian, not otherwise specified; Polynesian, not otherwise specified; Tahitian, Samoan, Tongan, Melanesian, not otherwise specified; Fiji Islander; New Guinean; other Asian, including Asian, not otherwise specified, and Oriental, not otherwise specified; and Pacific Islander, not otherwise specified.

^b^ Metropolitan was defined as having a population of 250 000 or greater; smaller metropolitan, 20 000 to less than 250 000.

**Table 4.** Factors Associated With Increased or Decreased RT Use by Age Group

RT Use	Age 18-39 y (n = 1280)	Age 40-64 y (n = 3937)	Age ≥65 y (n = 3736)
Increased	Private insurance; deep depth of invasion; larger tumor	Private insurance; deep depth of invasion; larger tumor; urban living location; transition of care; stage II; positive surgical margins	Private insurance; deep depth of invasion; larger tumors; urban living location; transition of care; undifferentiated grade; clinical stage II tumor; positive surgical margins
Decreased	White Hispanic race/ethnicity; distance to reporting facility	White Hispanic race/ethnicity; distance to reporting facility	1 Comorbidity; lower limb tumor; distance to reporting facility

Abbreviation: RT, radiation therapy.

### Multivariable Analysis on Treatment Type

Young adult patients were more likely to receive chemotherapy than older patients (ages 40-65 years: OR, 0.52; 95% CI, 0.45-0.60; *P* = .001; ≥65 years: OR, 0.16; 95% CI, 0.12-0.20; *P* = .001) (Table 5). Conversely, young adults were less likely to receive RT compared with older patients (40-65 years: OR, 1.40; 95% CI, 1.22-1.61; *P* = .001; ≥65 years: OR, 1.33; 95% CI, 1.10-1.61; *P* = .003) (Table 3). Timing of radiation (postoperative vs preoperative radiation) was not significant (Table 5). There was no statistical difference in the likelihood of amputation vs LSS for young compared with older adults (≥65 years: odds ratio [OR], 1.49; 95% CI, 1.00-2.23; *P* = .05) (Table 5).

**Table 5.** Multivariable Logistic Regression: Association of Age With Treatments^a^

Treatment	OR (95% CI)	*P* value
Chemotherapy vs none		
18-39 y (n = 1243)	1 [Reference]	NA
40-64 y (n = 3828)	0.52 (0.45-0.60)	.001
≥65 y (n = 3630)	0.16 (0.12-0.20)	.001
LSS vs amputation		
18-39 y (n = 521)	1 [Reference]	NA
40-64 y (n = 1218)	1.20 (0.90-1.60)	.21
≥65 y (n = 1367)	1.49 (1.00-2.23)	.05
Preoperative vs postoperative RT		
18-39 y (n = 759)	1 [Reference]	NA
40-64 y (n = 2710)	1.12 (0.92-1.35)	.26
≥65 (n = 2368)	0.90 (0.69-1.17)	.44

Abbreviations: LSS, limb sparing surgery; OR, odds ratio; RT, radiation therapy.

^a^ The following variables were included for adjustment: sex, race/ethnicity, insurance status, median income of patient’s zip code, education level in patient’s zip code, living location, distance to hospital, comorbidities, transition in care, primary site, grade, tumor size, tumor stage, depth of extension, and surgical margin.

## Discussion

In this analysis comparing sarcoma treatment across age groups in the NCDB, our results revealed significant differences wherein young adult patients were less likely to be treated with RT but more likely than other age groups to incorporate chemotherapy in the treatment regimen. The patient population in the young adult group were more likely to have tumors smaller than 5 cm and moderately differentiated tumor types compared with the older treatment groups. These findings suggest that the disease may not be as aggressive in the younger population. However, these findings are complicated by the fact that more young adults than older adults underwent amputation, with no significant difference in the likelihood of LSS with or without the use of radiation.

A significant difference also existed in the financial and demographic factors. Young adult patients were more likely to be medically uninsured, have a median income of less than $38 000, and lack a high school education. The NCDB data also showed that young adults were proportionally less likely to identify as races/ethnicities other than White and Hispanic, with greater percentages of Black and White Hispanic patients. A higher incidence rate of soft-tissue sarcoma among Black and White Hispanic individuals is consistent with other studies.^14,15^ These findings shed light on broader potential disparities identified in other analyses, such as one examining the Surveillance, Epidemiology, and End Results (SEER) and the Texas Cancer Registry (TCR) databases, which found higher mortality among Hispanic and non-Hispanic Black patients.^16^

There were observed similarities across the groups, including the predominance of primary tumors occurring in the lower limbs. In this analysis of the NCDB, we found no significant differences in clinical tumor stage across the age groups or in the use of preoperative or postoperative radiotherapy. The data set did not parse out different histology types or adverse events, which makes a thorough assessment of treatment across age groups more difficult to analyze against anecdotal or single-center results seen by clinicians.

### Limitations

This study has limitations. Most prominently, the nature of a retrospective study underscores the possibility of selection bias. While our analysis does indicate certain trends, such as less radiotherapy in the young adult population, it is difficult to tell whether these are indicators of less-aggressive tumors or decision-making adapted to younger ages. There is also a bias of chemotherapy being used in the younger population because of the chemosensitive nature of tumors with higher prevalence in this age group, such as Ewing sarcoma or high-grade osteosarcoma.^5^ Also, comorbidities could affect which treatment options are offered to patients, and in our study, older age groups had more comorbidities than younger groups. Previous studies using other cancer registries have sought to address questions on disparities in younger patient survival through an assessment of race and outcomes, including 5-year survival.^16^ These outcomes would represent areas of future exploration, in conjunction with inclusion of other databases, such as SEER and TCR as well as expansion to include pediatric cases, to further elucidate potential differences in treatment regimens across age lines.

## Conclusions

In this study, age groups were not significantly associated with the decision to use LSS; however, they did appear to be associated with the decision to use chemotherapy and RT. The young adult population was significantly less likely to receive RT and more likely to receive chemotherapy despite controlling for clinical and demographic factors. Further study is warranted to identify the clinical outcomes of these practice disparities.
